# Pneumatized superior turbinate as a cause of headache

**DOI:** 10.1186/1746-160X-3-3

**Published:** 2007-01-09

**Authors:** Elias Homsioglou, Dimitrios G Balatsouras, Gregory Alexopoulos, Antonis Kaberos, Michael Katotomichelakis, Vassilios Danielides

**Affiliations:** 1 Department of Otolaryngology, Medical School, Democritus University of Thrace. Dragana, Alexandroupolis, Greece; 2 Department of Otolaryngology, Tzanion General Hospital, 1 Afentouli & Zanni, Piraeus, Greece; 3 Department of Otolaryngology, "Agia Olga" General Hospital of Athens, 3-5 Agias Olgas, N. Ionia, Athens, Greece

## Abstract

**Background:**

A pneumatized superior turbinate is a rare cause of headache. Nasal endoscopy alone, does not provide us with adequate information for this inaccessible area of the superior nasal cavity. A coronal computed tomography (CT) must be obtained to confirm the diagnosis.

**Case presentation:**

We present a 40-year-old female with migraine-type headache and nasal obstruction. Nasal endoscopy revealed a mild septal deviation, a right middle concha bullosa and a paradoxically curved middle turbinate on the left side. Coronal CT-scan showed also the presence of a superior concha bullosa on the left, which was in close contact with the nasal septum. The patient underwent septoplasty and bilateral endoscopic sinus surgery, including partial removal of both the pneumatized middle turbinates in conjunction with gentle lateralization and resection of the lower half of the left superior turbinate. Prompt relief from headache and nasal symptoms was obtained.

**Conclusion:**

Pneumatized superior concha causing migrainous headache is a rare finding. Endoscopic surgery may provide permanent relief of symptoms.

## Background

The otorhinolaryngologic causes of headaches in most cases may be included in one of the following categories [1]: (1) Headaches associated with various sinus problems, most commonly acute or chronic rhinosinusitis; (2) headaches clearly connected to specific, easily recognizable nonsinus causes, such as neuralgias, migraine, otalgia, temporomandibular joint disease or vascular headaches; (3) non typical headaches, in patients with absence of sinus pathology. In the last group, midface discomfort, presenting as either pressure, fullness or even intense pain is a common occurrence. Its source may be dental, neural or nasal, presenting thus a challenge to the rhinologist.

Nasal causes of headaches include deviated nasal septum, engorgement of the turbinates, nasal neoplasm, pneumatized agger nasi cells, unusual deflections of uncinate process, paradoxically bent middle turbinate and variations of ethmoid bulla [2]. Pneumatized turbinates have been reported as rare causes of headache that deserve further evaluation [3,4].

The aim of this study is to present a rare case of a patient with headache of nasal origin, owed to the presence of enlarged pneumatized superior turbinate.

## Case presentation

A 40-year-old female, non smoker was referred by her family physician with migraine-type headache complaints and mild nasal congestion. The pain was located over the forehead and behind the left eye. Headaches occurred once every 3 weeks, triggered by weather and barometric changes with mild improvement during the menstruation. The duration of the headache was 3 days approximately and the pain was not associated with nausea, photophobia, vertigo or tinnitus. Its intensity was 9 on a 0–10 visual analogue scale.

The patient reported nephrotic syndrome at the age of 3, for which she had been on prednizolone for 10 years. A history of recurrent episodes of rhinosinusitis was also obtained.

**Figure 1:**
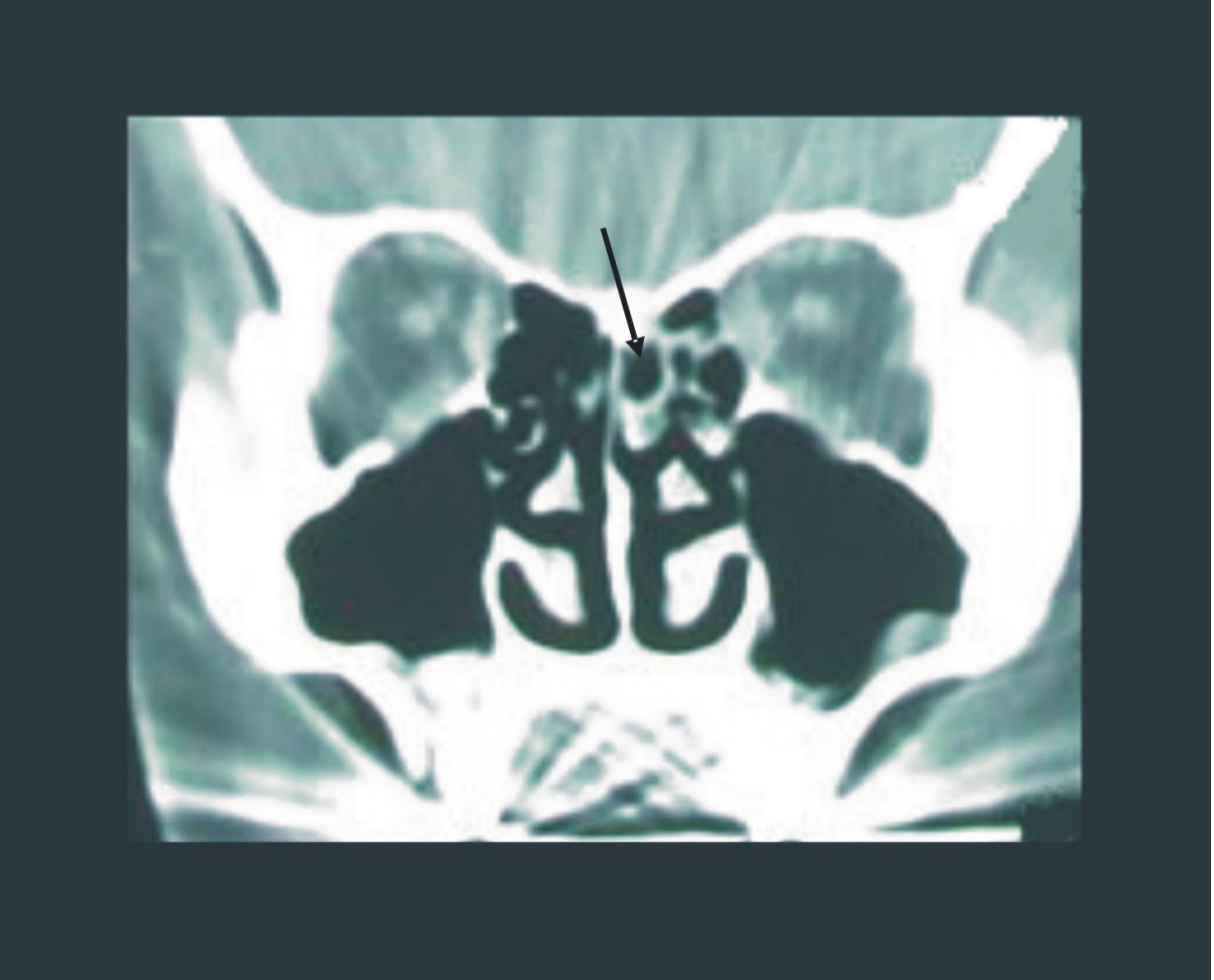
Coronal CT scan revealing pneumatization of the superior left turbinate (arrow).

Nasal endoscopy revealed a mild septal deviation, a right concha bullosa of the middle turbinate and a paradoxically curved middle turbinate on the left side. Coronal computed tomography (CT) scan confirmed the above findings and additionally revealed a superior concha bullosa and mild mucosal disease on the left side (Fig. 1).

The patient underwent a detailed laboratory examination to exclude any other possible causes of headache. Whole blood cell count, urinalysis, thyroxin level, biochemical tests, tumor markers, as well as Mantoux test and monotest were normal. Chest radiography, electrocardiography, and ultrasonography of the thyroid gland were also normal. Biochemical and serologic tests of hepatic dysfunction were negative. Serologic tests of complement fixation titers of the plasma antibodies against various common viruses were performed and were found normal as well.

**Figure 2:**
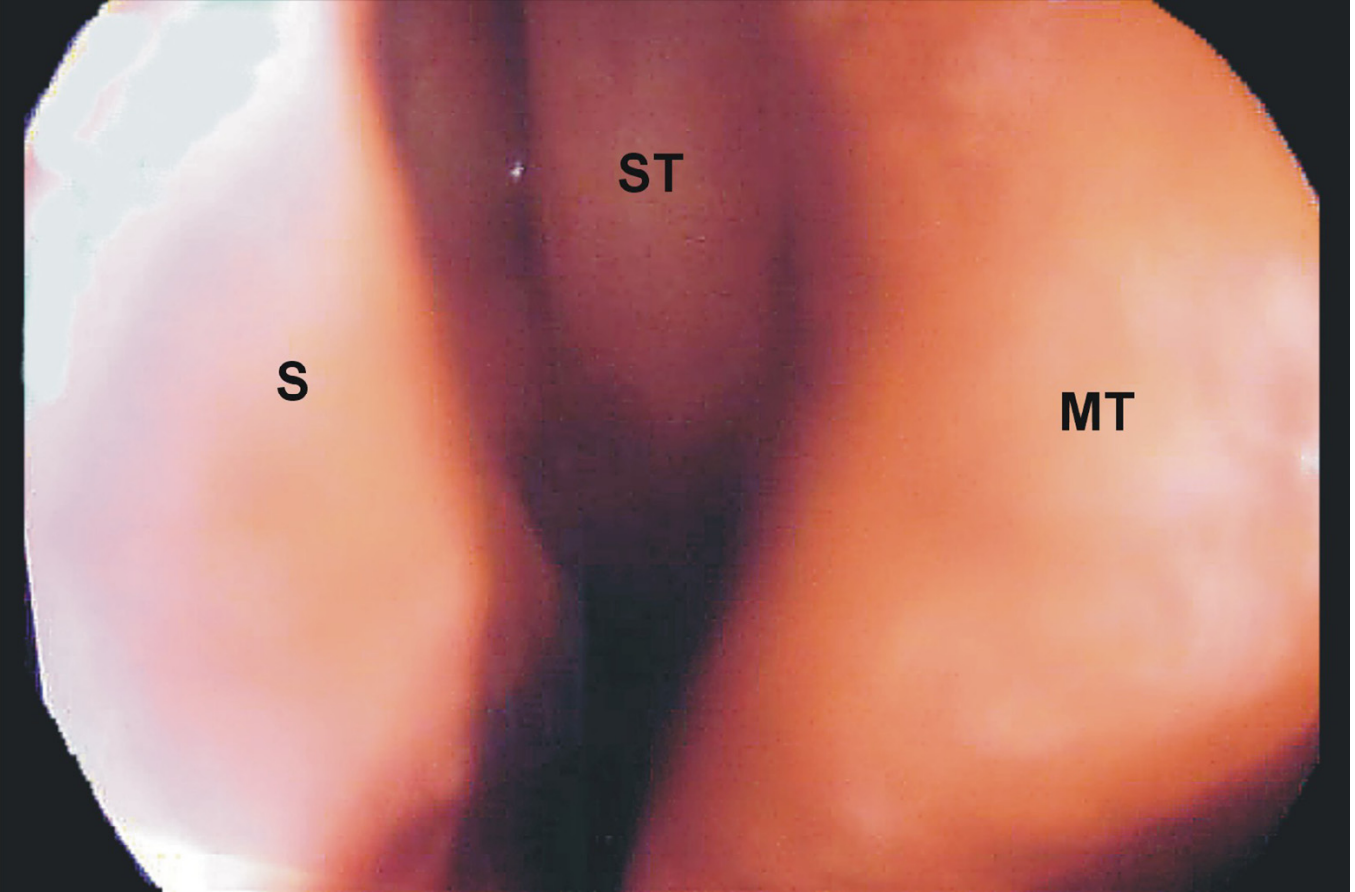
Endoscopic view of the left nasal cavity (ST: superior turbinate; S: septum; MT: middle turbinate).

The patient underwent nasal rigid endoscopy after application of local anesthetic and decongestant, which revealed the presence of a large superior concha in close contact with the nasal septum (Fig. 2). Direct application of local anesthetic (1% tetracaine hydrochloride solution) at the point of contact between the superior concha and the nasal septum improved headache by 4 points (score 5 at the scale of 0–10). Injection of local anesthetic (0.5 ml of 2% xylocaine with epinephrine 1:100.000) into the superior turbinate under endoscopic visualization eliminated the pain completely.

The patient was given endonasal steroids for 20 days without improvement. Then she was advised surgical intervention which she initially denied. The patient was followed for 3 months without any improvement and she, finally, decided to undergo surgery. Septoplasty and bilateral endoscopic sinus surgery was performed, including partial removal of both the pneumatized middle turbinates in conjunction with gentle lateralization and resection of the lower half of the left superior turbinate. The interior of the superior turbinate was found devoid of fluid or any other content. The patient reported prompt relief of her headache and at 13 months of follow-up postoperatively she remains free from headache or any other nasal symptoms. Although the patient did never report anosmia, she was examined by using the "Sniffin' sticks" test 1-year-postoperatively [5]. The examination showed that odor threshold, odor discrimination and odor identification were within normal limits. It appears, thus, that although part of the olfactory mucosa might had been removed by the partial resection of the superior turbinate, sufficient olfactory epithelium had remained intact, resulting in preservation of normal olfactory function.

Although pneumatization of the middle nasal turbinate has been occasionally reported [6], pneumatization of the superior and inferior turbinates are very rare findings [4,7]. The presence of a superior concha bullosa is not always recognizable with nasal endoscopy alone, due to the minimally accessible area of the upper nasal cavity. For this reason, the superior nasal turbinate has been called the forgotten turbinate [8]. Coronal CT-scan provides useful information for this inaccessible area.

Association of a massive extensively pneumatized superior turbinate with headache is very rare. In these cases the superior turbinate is forced anteriorly and inferiorly at the area between the middle turbinate and the nasal septum, leading to intranasal mucosal contact [9]. Although the phenomenon of referred headache owed to intranasal mucosal contact was recognized as early as 1888 by Roe [10] and has been since, occasionally, reported, with the advent of functional endoscopic sinus surgery and CT imaging, resurgence of interest in headache of nasal origin has been observed. Stammberger and Wolf [1] reviewed the possible mechanisms involved in the genesis of referred headaches in the nasal area. According to them, afferent fibers from pain receptors in the nasal mucosa terminate in the same group of sensory neurons in the sensory nucleus of the trigeminal nerve, as fibers innervating cutaneous receptors, located at several peripheral segmental dermatomes of the ophthalmic and maxillary divisions of the trigeminal nerve. These two common pathways converge along the same final neurons to a common area of the cortex. Accordingly, the cortical center can not distinguish the original peripheral source of the pain impulses and they may be misinterpreted as coming from other skin areas, such as the temple, the zygoma or the forehead. The pain may be perceived also from other end-organs innervated by terminal branches within the trigeminal system, such as dura, intracranial and scalp vessels or the eye [8].

An important issue in the generation of headaches of nasal origin is mediation of neuropeptides, such as substance P [1,8,11]. These may be released by mechanical pressure induced in areas of contacting opposing mucosal surfaces, such as the superior turbinate and the nasal septum. Substance P can be liberated at both the central and the peripheral ends of a sensory neuron, mediating not only central pain reflexes, via afferent C fibers, but at the same time local reflexes at the nasal mucosa, resulting from reverse impulses and manifestating as vasodilation, extravasation of plasma, hypersecretion, and smooth muscle contraction. This axon reflex can explain why an initial small localized lesion, such as a limited mucosal lesion or area of nasal mucosal contact, may trigger severe long-standing headaches, frequently projecting to different areas of the head.

Many areas of the nose and the paranasal sinuses have been implicated as causes of referred headaches, including superior concha bullosa as a rare occurrence. Clerico [8] was the first who suggested the superior turbinate as a source of referred headache, with features consistent of common migraine. Two more reports on this issue followed [7,9]. Clerico [8] proposed that before surgery, the application of local anesthetic and/or decongestant (diagnostic block) on the area of the superior turbinate, under endoscopic visualization, might confirm this anatomic variation as the source of headache. We performed this test, and although significant reduction of the pain was obtained, the pain was not completely eliminated. This was probably due to the fact that local decongestants and anesthetic sprays are deposited primarily in the anterior half of the nasal cavity and may not adequately reach the superior turbinate. Complete relief of headache was obtained only after injection of local anesthetic into the superior turbinate under endoscopic guidance. The positive finding of this diagnostic test and resolution of the symptoms after surgical intervention prove the association of our patient's headache with the presence of the massive superior concha bullosa.

Finally, the presence of a nasal osteoma as a rare cause of headaches of nasal origin should be mentioned. Paranasal sinus and intranasal osteomas are histologically benign and slow-growing tumors, which are usuallly asymptomatic, but when they enlarge they may produce pressure symptoms such as headaches. They occasionally cause obstruction or infection and may grow into the orbit or intradurally causing neurological manifestations. Paranasal sinus osteomas usually occur in the frontal sinus and less frequently in the other sinuses [12]. A few cases of intranasal osteomas were also reported, mainly in the middle turbinate and once in the superior turbinate [13]. All cases of turbinal osteomas were characterized by accompanying headache, probably due to the same pathogenetic mechanisms implicated in headache caused by the presence of a pneumatized superior concha, as previously mentioned. CT scan may assist in differential diagnosis by depicting the lesion and providing detailed information concerning the bony structures of the region.

## Conclusion

Pneumatized superior concha is a rare anatomic variant that is usually combined with other anatomic abnormalities of the nasal cavity and the lateral nasal wall. Resulting nasal mucosal contact may cause migraine-type headache, even without evidence of mucosal disease. The diagnosis of a pneumatized superior concha is based primarily on CT scans, because this area is usually inaccessible through nasal endoscopy. Endoscopic surgery may provide permanent relief of the headache.

## Competing interests

The author(s) declare that they have no competing interests.

## Authors' contributions

EH diagnosed and treated the patient and drafted the manuscript. DGB   diagnosed and treated the patient and assisted in drafting the manuscript; GA assisted in the diagnostic work-up of the patient; AK assisted in the diagnostic work-up of the patient; MK examined the patient and assisted in drafting the manuscript. VD has been involved in revising the manuscript critically for important intellectual content.

All authors read and approved the final manuscript.
